# Comparison of Perioperative, Renal Functional, and Oncological Outcomes Between Off-Clamp and On-Clamp Robot-Assisted Partial Nephrectomy for Renal Tumors: An Updated Evidence-Based Analysis

**DOI:** 10.3389/fonc.2021.730662

**Published:** 2021-09-21

**Authors:** Yin Huang, Dehong Cao, Zeyu Chen, Bo Chen, Jin Li, Jianbing Guo, Qiang Dong, Qiang Wei, Liangren Liu

**Affiliations:** ^1^Department of Urology/Institute of Urology, West China Hospital, Sichuan University, Chengdu, China; ^2^West China School of Medicine, Sichuan University, Chengdu, China

**Keywords:** robot-assisted partial nephrectomy, off-clamp, on-clamp, kidney cancer, renal function

## Abstract

**Objectives:**

We aimed to report the latest and largest pooled analysis and evidence update to compare the perioperative, renal functional, and oncological outcomes between off-clamp and on-clamp robot-assisted partial nephrectomy (RAPN) for renal tumors.

**Patients and methods:**

We performed a systematic literature search using PubMed, Embase, and Web of Science up to August 2021 for studies that compared the efficacy and/or safety between off-clamp and on-clamp RAPN for renal tumors. Outcomes measured were operating time, estimated blood loss (EBL), conversion rate, length of stay (LOS), complication rate, transfusion rate, long-term % decrease in estimated glomerular filtration rate (eGFR), positive surgical margin rate, and recurrence rate.

**Results:**

A total of 21 eligible articles involving 4,493 patients (1,274 off-clamp *versus* 3,219 on-clamp) were included for the evidence synthesis. Baseline characteristics of the two groups were similar in all outcomes except that lower R.E.N.A.L. score and smaller tumor size were observed in the off-clamp group. Pooled analysis showed shorter operative time, higher EBL, and lower complication rate in the off-clamp group. No significant difference was observed in the conversion rate, LOS, and transfusion rate. The recurrence rates were similar in the two groups, while a lower positive surgical margin rate was observed in the off-clamp group. Finally, the off-clamp group had a superior postoperative renal functional outcome.

**Conclusions:**

Given the presence of heterogeneity and potential bias, urologists should select the clamp strategy based on their experience and patient-specific factors.

## Introduction

Renal cancer is one of the most common malignant tumors, with an estimated 73,750 new cases and 14,830 deaths in the USA in 2020 (1). In the past few years, partial nephrectomy (PN) has been considered as the standard surgical procedure for cT1 (<7 cm) renal tumors due to its equivalent oncological outcomes, better preservation of renal function, and superior overall survival compared with radical nephrectomy (2, 3). As a minimally invasive operation, robot-assisted PN (RAPN) is being increasingly performed globally, which has superiority in dissection and intracorporeal suturing (4, 5). As we all know, the major goals of PN are to control tumors, avoid intraoperative and postoperative complications, and preserve renal function (6). Three factors have been validated associated with postoperative renal function, including preoperative renal function, quantity of preserved renal parenchyma, and warm ischemia time (WIT), of which WIT was regarded as a major modifiable factor for renal function preservation (7).

In recent years, under the condition of more and more surgeons performing the RAPN with zero ischemia technique, namely, the off-clamp approach to minimize the WIT (8), plenty of studies have been conducted to identify whether the off-clamp RAPN is superior to the on-clamp in efficacy and safety, especially in postoperative renal function preservation (9–29). However, consensus of which clamping technique in RAPN is optimal with respect of perioperative, renal functional, and oncological outcomes remains controversial.

There were two published meta-analyses that compared the efficacy and safety of on-clamp and off-clamp RAPN, which both did not assert that the off-clamp approach is the optimal clamping technique in RAPN (30, 31). Whereafter, six novel original studies of the same topic have been published during 2019–2020 (24–29). Thus, we reported a pooled analysis and evidence update to compare the perioperative, renal functional, and oncological outcomes between off-clamp and on-clamp RAPN for renal tumors.

## Materials and Methods

### Literature Search

The present evidence-based analysis was conducted following the PRISMA (Preferred Reporting Items for Systematic Reviews and Meta-Analysis) 2020 statement (32) and was prospectively registered in the PROSPERO (CRD42021228512). The PRISMA 2020 checklist is shown in **Supplementary Table S1**. We performed a systematic literature search using PubMed, Embase, and Web of Science up to August 2021 for studies that compared the efficacy and/or safety between off-clamp and on-clamp RAPN for renal tumors and published in English. We searched the databases using the following terms: “robot-assisted”, “robotic-assisted”, “robot”, “robotic”, “partial nephrectomy”, “nephron sparing surgery”, “clamp”, “clamping”, “off-clamp”, and “on-clamp”. The detailed search strategy is presented in **Supplementary Table S2**. In addition, the reference lists of all eligible studies were manually reviewed. Two investigators searched and evaluated the included studies independently. Any disagreement in literature search was resolved by consensus.

### Identification of Eligible Studies

Studies were included if they met the following criteria: (1) the study design was randomized controlled, cohort, or case–control; (2) studies were conducted in adults with renal tumors; (3) studies comparing off-clamp RAPN with on-clamp RAPN; (4) at least one perioperative (operating time, estimated blood loss (EBL), conversion rate, length of stay (LOS), complication rate, and transfusion rate), renal functional (postoperative estimated glomerular filtration rate (eGFR) decrease and serum creatine increase during follow-up), or oncological (positive surgical margins rate and recurrence rate) outcome was evaluated; and (5) sufficient data to calculate odds ratio (OR) or weighted mean difference (WMD).

We excluded reviews, letters, editorial comments, case reports, conference abstracts, pediatric articles, unpublished articles, and non-English articles. We defined the off-clamp RAPN as the RAPN performed without any hilar clamping procedure, and the on-clamp RAPN was identified as clamping the main renal artery during the entire procedure. Thus, studies that focused on selective-clamp RAPN, super-selective clamp RAPN, and early-unclamping RAPN were also excluded.

### Data Extraction

Data extraction was performed by two investigators independently. Any disagreement was resolved by the third investigator to make a final decision. We extracted the following data from included studies: first author, publication year, study period, country of study, study design, sample size, age, body mass index (BMI), American Society of Anesthesiologist (ASA) score, preoperative eGFR, preoperative serum creatinine, tumor size, R.E.N.A.L. score, follow-up time, operating time, EBL, conversion rate, LOS, complication rate, transfusion rate, long-term (postoperative 6 months or longer) % decrease in eGFR, positive surgical margin rate and recurrence rate. When continuous variables in the study were reported as median with range or interquartile range, we calculated the mean ± standard deviation through the validated mathematical method (33, 34). When data were missing or not reported in the study, we contacted the corresponding authors to obtain completed data if available.

### Quality Assessment

The Newcastle–Ottawa Scale (NOS) was used for evaluating the quality of included studies (35), and studies with seven to nine points were regarded as high quality (36). In addition, we assessed the level of evidence for each study according to the Oxford Centre for Evidence-Based Medicine Levels of Evidence Working Group (37). Two investigators independently evaluated the quality and level of evidence for eligible studies, and any discrepancy was resolved through discussion.

### Statistical Analysis

Evidence synthesis was performed in Review Manager 5.3 version (Cochrane Collaboration, Oxford, UK). The WMD and OR were applied for the comparison of continuous and dichotomous variables, respectively. All metrics were reported with 95% confidential intervals (CIs). The heterogeneity in studies was assessed through the chi-squared (χ^2^) test (Cochran’s *Q*) and inconsistency index (*I* ^2^) (38). χ^2^ *p* value < 0.05 or *I* ^2^ > 50% were considered as significant heterogeneity. A random-effect model was used to estimate the combined WMD or OR when significant heterogeneity was detected (χ^2^ *p* value < 0.05 or *I* ^2^ > 50%). Otherwise, the fixed-effect model was applied. In addition, we performed one-way sensitivity analyses to evaluate the effect of included studies on the combined results for outcomes with significant heterogeneity. Publication bias was evaluated visually by creating funnel plots *via* Review Manager 5.3 version (Cochrane Collaboration, Oxford, UK), as well as by conducting Egger’s regression tests (39) using Stata 12.0 version (Stata Corp, College Station, TX, USA) for outcomes with 10 or more included studies. *p* value < 0.05 was considered as statistically significant publication bias.

## Results

### Literature Search and Study Characteristics

**Figure 1:**
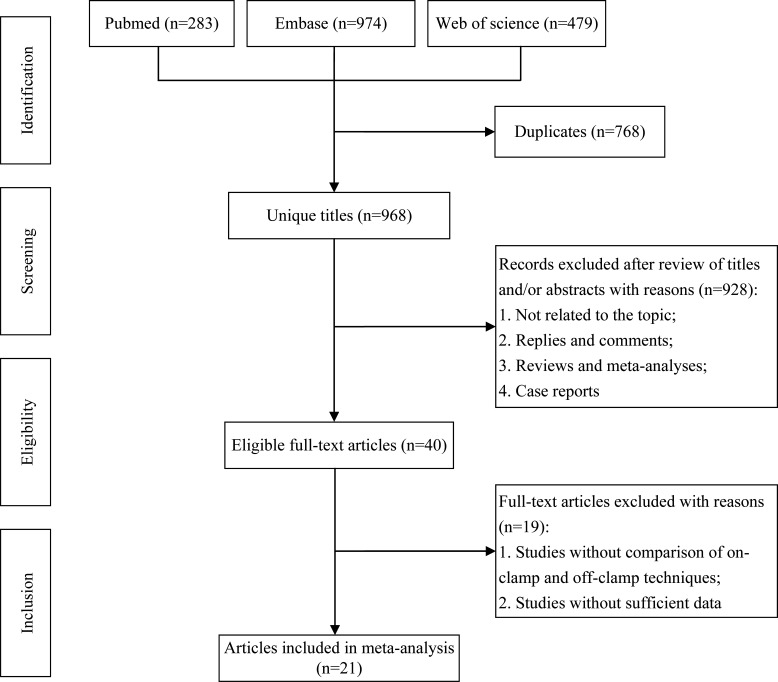
Flowchart of the systematic search and selection process.

The flowchart of the systematic search and selection process is presented in **Figure 1**. A total of 1,736 relevant articles in PubMed (n = 283), Embase (n = 974), and Web of Science (n = 479) were yielded through systematic literature search. After removing duplicate papers, 968 titles and abstracts were reviewed. Finally, 21 full-text articles involving 4,493 patients (1,274 off-clamp *versus* 3,219 on-clamp) were included for the pooled analysis (9–29). Of these articles, 7 were prospective cohort studies (9, 10, 12, 13, 16, 18, 25, 27), 10 were retrospective cohort studies (11, 14, 15, 17, 19–21, 23, 24, 29), and 3 were prospective randomized studies (22, 26, 28). **Table 1** shows the characteristics, level of evidence, and quality score of each included study. The median (range) quality score was 7 (5–8), and 15 studies were identified as high quality (9, 11, 12, 14, 16–24, 28, 29). The details of quality assessment of all eligible studies are presented in **Supplementary Table S3**.

**Table 1:** Baseline characteristics of include studies and methodological assessment.

Authors	Study period	Country	Study design	Patients (n)	Median follow-up (months)	Level of evidence	Quality score
				Off-clamp/on-clamp			
White et al. (9)	2008	USA	Prospective	8/20	15	2b	8
Novak et al. (10)	2009–2010	USA	Prospective	22/35	–	4	6
Tanagho et al. (11)	2008–2011	USA	Retrospective	29/29	9	2b	7
Kaczmarek et al. (12)	2007–2011	USA	Prospective	49/283	13.5	2b	8
Krane et al. (13)	2010-2011	USA	Prospective	19/18	3.8	2b	6
Acar et al. (14)	2010–2013	Turkey	Retrospective	30/14	18.9	2b	8
Komninos et al. (15)	2007–2013	Korea	Retrospective	23/114	–	4	6
Ener et al. (16)	2009–2015	Turkey	Prospective	12/22	–	2b	7
Peyronnet et al. (17)	2010–2014	France	Retrospective	26/104	12	2b	8
Rosen et al. (18)	2008–2016	USA	Prospective	41/82	9.2	2b	7
Anderson et al. (19)	2009–2015	USA	Retrospective	50/50	9	2b	7
Mari et al. (20)	2011–2014	Italy	Retrospective	120/120	40	2b	8
Taweemonkongsap et al. (21)	2010–2016	Thailand	Retrospective	12/27	18	2b	8
Anderson et al. (22)	2013–2017	USA	Prospective	40/30	3	1b	7
Bertolo et al. (23)	2007–2017/2010–2017	USA/Italy	Retrospective	200/400	–	2b	7
Guo et al. (24)	2015–2017	China	Retrospective	48/45	12	2b	8
Anceschi et al. (25)	2013–2019	USA/Italy	Prospective	27/27	13	2b	6
Antonelli et al. (26)	2014–2018	Italy	Prospective	91/129	–	1b	6
Beksac et al. (27)	2006–2018	USA	Prospective	39/375	–	2b	5
Antonelli et al. (28)	2015–2018	Italy	Prospective	164/160	–	1b	7
Mellouki et al. (29)	2011–2019	France	Retrospective	224/1135	38	2b	7

### Demographic Characteristics

There were no significant differences among the two groups in terms of age (WMD: -0.16; 95% CI: -0.73, 0.41; *p* = 0.58), gender (male/total, OR: 0.99; 95% CI: 0.85, 1.15; *p* = 0.87), BMI (WMD: 0.19; 95% CI: -0.18, 0.56; *p* = 0.32), ASA score (WMD: 0.02; 95% CI: -0.08, 0.13; *p* = 0.66), preoperative eGFR (WMD: 0.89; 95% CI: -0.33, 2.11; *p* = 0.15), and preoperative serum creatine (WMD: -0.01; 95% CI: -0.06, 0.03; *p* = 0.60). However, the two groups were significantly different in baseline characteristics in terms of R.E.N.A.L. score (WMD: -0.55; 95% CI: -0.93, -0.17; *p* = 0.004) and tumor size (WMD: -0.37; 95% CI: -0.67, -0.08; *p* = 0.01) (**Table 2**).

**Table 2:** Demographics and clinical characteristics of included studies.

Outcomes	Studies	No. of patients	WMD or OR	95% CI	p-value	Heterogeneity			
		Off-clamp/On-clamp				Chi^2^	df	p-value	*I*^2^ (%)
Age (years)	(19)	1,129/3,107	-0.16	[-0.73, 0.41]	0.58	15.61	18	0.62	0
Gender (male)	(17)	670/1,568	0.99	[0.85, 1.15]	0.87	17.48	16	0.87	8
BMI (kg/m^2^)	(16)	952/2,309	0.19	[-0.18, 0.56]	0.32	10.01	15	0.82	0
ASA score	(6)	263/582	0.02	[-0.08, 0.13]	0.66	3.42	5	0.63	0
R.E.N.A.L. score	(17)	1,101/2,967	-0.55	[-0.93, -0.17]	0.004*^a^*	106.75	16	<0.00001	85
Tumor size (cm)	(19)	1,199/2,842	-0.37	[-0.67, -0.08]	0.01*^a^*	238.44	18	<0.00001	92
Preoperative eGFR (mL/min/1.73 m^2^)	(17)	968/1,925	0.89	[-0.33, 2.11]	0.15	60.37	16	<0.00001	73
Preoperative sCr (mg/dL)	(6)	229/251	-0.01	[-0.06, 0.03]	0.60	5.50	5	0.36	9

^a^Statistically significant.

BMI, body mass index; ASA, American Society of Anesthesiologists; eGFR, estimated glomerular filtration rate; sCr, serum creatine; WMD, weighted mean difference; OR, odds ratio; CI, confidence interval.

### Operating Time

**Figure 2:**
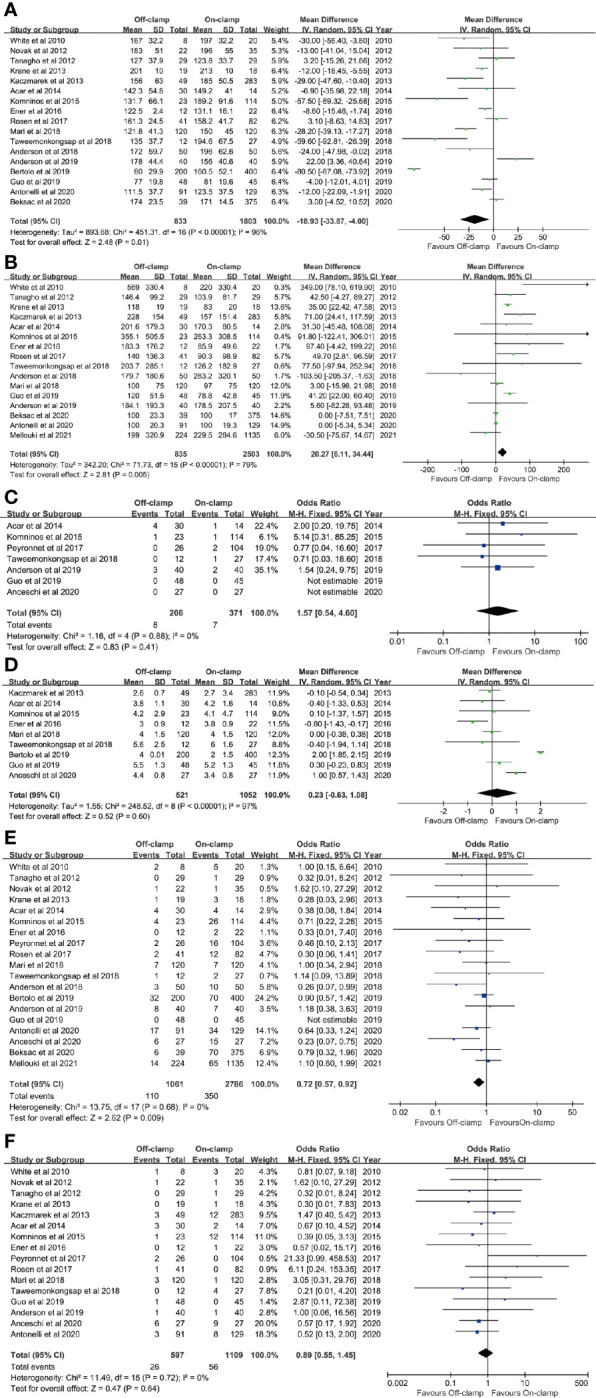
Forest plots of perioperative outcomes: **(A)** operating time, **(B)** estimated blood loss, **(C)** conversion rate, **(D)** length of stay, **(E)** complication rate, and **(F)** transfusion rate.

**Figure 3:**
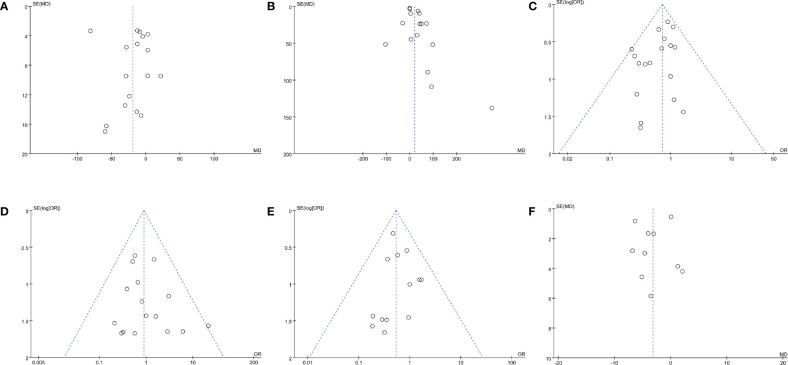
Funnel plots of **(A)** operating time, **(B)** EBL, **(C)** complication rate, **(D)** transfusion rate, **(E)** positive surgical margins rate, and **(F)** long-term % decrease in eGFR.

Data of operating time were synthesized from 17 studies including 2,636 patients (833 off-clamp *versus* 1,803 on-clamp) (9–16, 18–24, 26, 27). Pooled analysis revealed a significant shorter operating time in the off-clamp group (WMD: -18.93; 95% CI: -33.87, -4.00; *p* = 0.01) with a significant heterogeneity (*I* ^2^ = 96%, *p* < 0.00001) (**Figure 2A**). A visual assessment of the funnel plot indicated the presence of slight publication bias (**Figure 3A**). However, Egger’s test was not statistically significant (*p* = 0.737).

### EBL

Analysis of EBL was conducted in 16 studies with 3,338 patients (835 off-clamp *versus* 2503 on-clamp) (9, 11–16, 18–22, 24, 26, 27, 29). Pooled analysis detected a significantly higher EBL in the off-clamp group (WMD: 20.27; 95% CI: 6.11, 34.44; *p* = 0.005) with a statistically significant heterogeneity (*I* ^2^ = 79%, *p* < 0.00001) (**Figure 2B**). Funnel plots revealed a slight publication bias (**Figure 3B**) while no statistically significant publication bias was detected through Egger’s test (*p* = 0.061).

### Conversion Rate

Seven studies involving 577 patients (206 off-clamp *versus* 371 on-clamp) were included in the analysis (14, 15, 17, 21, 22, 24, 25). Pooled results demonstrated that the rate of conversion to radical or open surgery was similar between the two groups (OR: 1.57; 95% CI: 0.54, 4.60; *p* = 0.41), and no significant heterogeneity was observed (*I* ^2^ = 0%, *p* = 0.88) (**Figure 2C**).

#### LOS

Nine articles reported the data of LOS, including 1,573 patients (521 off-clamp *versus* 1,052 on-clamp) (12, 14–16, 20, 21, 23–25). No significant difference was detected among the two groups (WMD: 0.23; 95% CI: -0.63, 1.08; *p* = 0.60), but statistically significant heterogeneity was observed (*I* ^2^ = 97%, *p* < 0.00001) (**Figure 2D**).

### Complication rate

Data of the complication rate (including intraoperative and/or postoperative complications) were available in 19 studies with a total of 3,847 patients (1,061 off-clamp versus 2,786 on-clamp) (9–11, 13–27, 29). Pooled analysis revealed a significantly lower rate of complication in the off-clamp group compared with the on-clamp group (OR: 0.72; 95% CI: 0.57, 0.92; *p* = 0.009) (**Figure 2E**). No significant heterogeneity (*I* ^2^ = 0%, *p* = 0.68) and statistical (Egger’s test, *p* = 0.067) or visual (**Figure 3C**) evidence of publication bias were detected.

### Transfusion Rate

There were 16 articles that reported the data of transfusion rate between the two groups, including 1,706 patients (597 off-clamp *versus* 1,109 on-clamp) (9–18, 20–22, 24–26). Evidence synthesis observed a similar transfusion rate in the two groups (OR: 0.89; 95% CI: 0.55, 1.45; *p* = 0.64) without significant heterogeneity (*I* ^2^ = 0%, *p* = 0.72) (**Figure 2F**) and statistical (Egger’s test, *p* = 0.368) or visual (**Figure 3D**) evidence of publication bias.

### Positive Surgical Margin Rate

**Figure 4:**
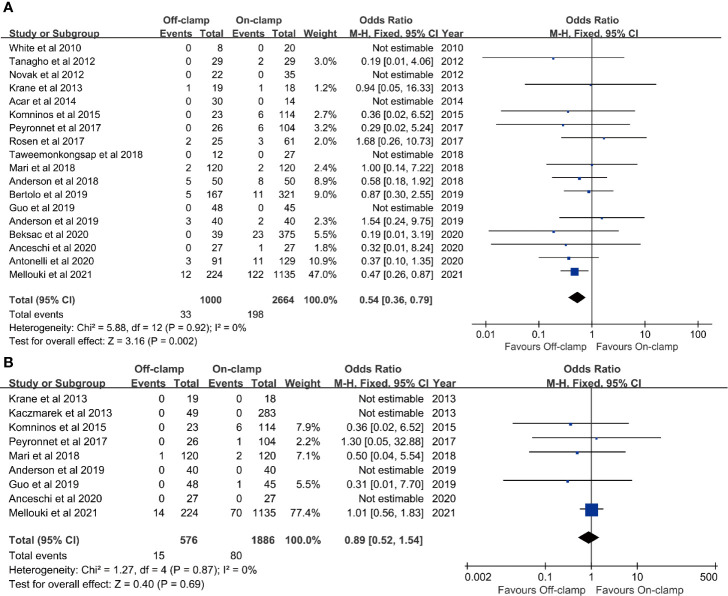
Forest plots of oncological outcomes: **(A)** positive surgical margins rate and **(B)** recurrence rate.

Eighteen studies with 3,664 patients (1,000 off-clamp *versus* 2,664 on-clamp) were included in the analysis for positive surgical margin rate (9–11, 13–15, 17–27, 29). Pooled analysis indicated that the off-clamp group had a significantly lower positive surgical margin rate (OR: 0.54; 95% CI: 0.36, 0.79; *p* = 0.002) (**Figure 4A**). No significant heterogeneity (*I* ^2^ = 0%, *p* = 0.92) and statistical (Egger’s test, *p* = 0.946) or visual (**Figure 3E**) evidence of publication bias were observed.

### Recurrence Rate

Data of recurrence rate were obtained from nine studies with 2,462 patients (576 off-clamp *versus* 1,886 on-clamp) (12, 13, 15, 17, 20, 22, 24, 25, 29). No significant difference was observed between the two groups for recurrence rate (OR: 0.89; 95% CI: 0.52, 1.54; *p* = 0.69), and no significant heterogeneity (*I* ^2^ = 0%, *p* = 0.87) was detected (**Figure 4B**).

### Long-Term % Decrease in eGFR

**Figure 5:**
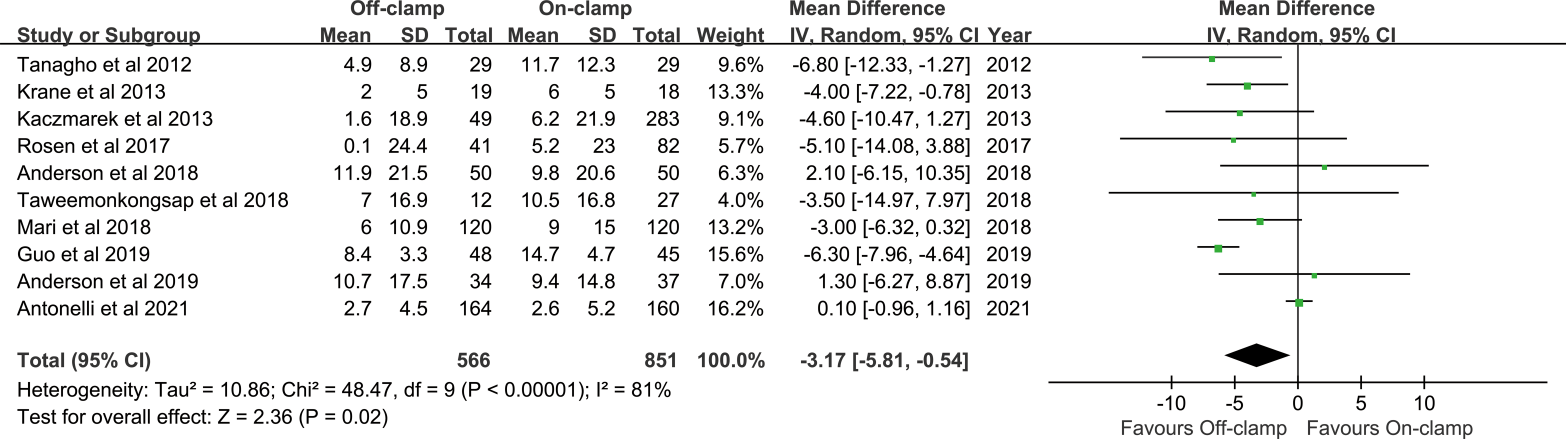
Forest plots of renal functional outcome: long-term % decrease in eGFR.

Ten articles were included in the analysis for long-term % decrease in eGFR, involving 1,417 patients (566 off-clamp *versus* 851 on-clamp) (11–13, 18–22, 24, 28). Evidence synthesis showed that the off-clamp group had a significantly lower long-term % decrease in eGFR (WMD: -3.17; 95% CI: -5.81, -0.54; *p* = 0.02) with a significant heterogeneity (*I* ^2^ = 81%, *p* < 0.00001) (**Figure 5**). Both funnel plot (**Figure 3F**) and Egger’s test (*p* = 0.423) did not detect publication bias.

### Sensitivity Analysis

**Figure 6:**
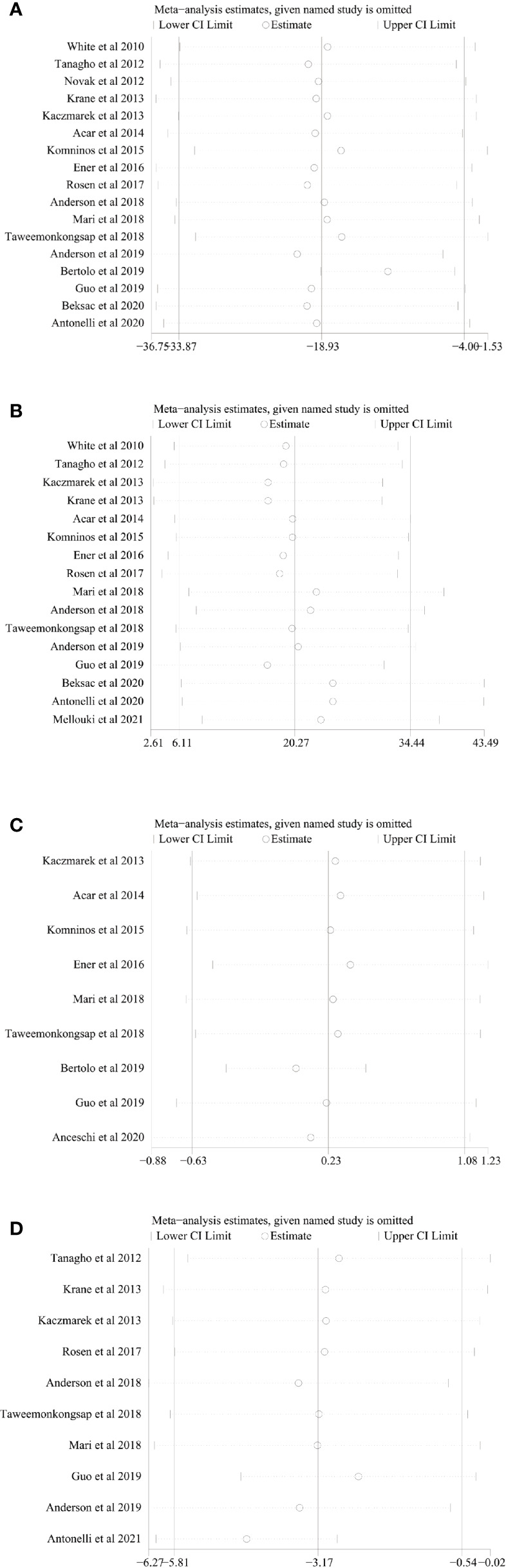
Sensitivity analysis of **(A)** operating time, **(B)** estimated blood loss, **(C)** length of stay, and **(D)** long-term % decrease in eGFR.

We conducted one-way sensitivity analyses for comparison of operating time, EBL, LOS, and long-term % decrease in eGFR to evaluate the influence of each individual study on the combined WMD through removing the individual study one by one. Sensitivity analyses revealed that the new combined WMD remained constant after exclusion of any individual study for operating time (**Figure 6A**), EBL (**Figure 6B**), LOS (**Figure 6C**), and long-term % decrease in eGFR (**Figure 6D**). However, when we excluded the data reported by Antonelli et al. in 2021 (28), the heterogeneity for the long-term % decrease in eGFR disappeared (*I* ^2^ = 21%, *p* = 0.26), suggesting that this study accounts for most of the heterogeneity.

## Discussion

At present, RAPN has been performed widely as a favorable surgical procedure for patients with localized renal tumors since its superiority in dissection, intracorporeal suturing, and preservation of renal function (40, 41). As we all know, there are three factors that have been regarded as major predictors of postoperative renal function: preoperative renal function, quantity of preserved renal parenchyma, and WIT (42). In 2009, White et al. firstly reported a comparative study of RAPN with or without clamping of the renal artery, which initially evaluated the efficacy and safety of the zero ischemia technique in RAPN (9). After that, numerous cohort studies that focused on the comparison of off-clamp RAPN with on-clamp RAPN have been published. However, the perioperative, oncological, and renal functional outcomes of the two clamping techniques in RAPN were still a matter of wide debate all over the world (14, 17, 22, 24, 30, 31). Under these conditions, we performed the latest and largest systematic review and pooled analysis of 21 comparative studies including 4,493 patients, and our results revealed several important findings.

First, results on perioperative outcomes showed a significantly shorter operating time in the off-clamp group, however, which may attribute to the selective bias that the off-clamp group had a lower R.E.N.A.L. score and smaller tumor size. Similarly, data from the CLOCK Randomized Clinical Trial indicated that the transition from off-clamp to on-clamp RAPN is also associated with renal mass diameter and complexity (43). In addition, shorter operative time in the off-clamp group was likely related to the lower complexity of tumors in the group and the avoidance of renal pedicle dissection in the technique (17, 23). Higher EBL was observed in the off-clamp group, reasonably due to the natural result of unclamping the renal vessels during the surgical procedure (12). Although the difference of EBL was statistically significant, its clinical relevance was limited since the transfusion rates were similar in the two clamping techniques. Moreover, we observed a lower complication rate in the off-clamp group. However, as mentioned previously, the difference may also be related to the smaller tumor size and the lower complexity of tumors in the off-clamp group (44). Patients who underwent on-clamp RAPN may be more technically challenging (19). Furthermore, conversion rates and LOS were similar in the two groups.

Second, analyses of the oncological outcomes in the two clamping techniques revealed a lower positive surgical margin rate in the off-clamp group but similar rates of recurrence in the two groups, which contradicts previous meta-analyses that did not observe a significant difference in the positive surgical margin rate (30, 31). However, the reason for these findings was still unclear. We might still assume that the complexity of tumors and clamping technique itself may influence the tumor dissection technique, leading to the different positive surgical margin rate in the two groups. On the other hand, a retrospective study reported by Shah et al. found that positive surgical margins after PN were associated with an increased risk of recurrence (45), while the relationship between positive surgical margins and recurrence after RAPN is still uncertain.

Third, a pooled analysis of renal functional outcome evaluated by a long-term % decrease in eGFR showed that the off-clamp group had a superior preservation of postoperative renal function. The present result was consistent with the finding of the meta-analysis reported by Cacciamani et al. (31), but contradicted the report of Antonelli et al. (30). Although there was significant heterogeneity in the long-term % decrease in eGFR, the difference remained significant when we excluded the main sources of heterogeneity (WMD: -4.48; 95% CI: -6.14, -2.82; *p* < 0.00001). However, it is worth noting that superior functional outcomes of the off-clamp group which are reported by previous observational studies (11, 13, 24) have not been confirmed in any RCTs (22, 28), indicating potential selection bias in our meta-analysis. Moreover, short-term renal functional outcomes after RAPN are still controversial (12, 15). Unfortunately, we failed to evaluate the short-term change in eGFR of the two groups since the deficiency of data and the data of change in serum creatine, which is another important measurement of postoperative change in renal function, were also insufficient to conduct pooled analysis.

Our study reported the latest and largest evidence-based analysis that directly and exclusively compared the perioperative, renal functional, and oncological outcomes of off-clamp and on-clamp RAPN in patients with renal tumors. However, we must acknowledge several limitations of the present study. Primarily, there were only three prospective randomized studies (9.5%) included in our pooled analysis. Most of the included studies were retrospective or prospective cohort design, without proper control of confounders. Furthermore, significant heterogeneity was observed in several outcomes including operating time, EBL, LOS, and long-term % decrease in eGFR. Although we performed sensitivity analyses to evaluate the stability of results, the derivation of heterogeneity was still unclear for several outcomes. Considering the potential confounders, results of the present pooled analysis should be interpreted with caution. Finally, we failed to provide a more comprehensive evaluation of the postoperative renal functional outcomes in the two groups due to the insufficient data of short-term change in eGFR and the increase of serum creatine after RAPN.

Notwithstanding several limitations of our study, we reported the latest and largest meta-analysis that added six novel articles (four cohort studies (24, 25, 27, 29) and two prospective randomized studies (26, 28) published during 2019–2021 on the bases of previous studies, which makes our evidence more credible. Our evidence-based analysis validated previous studies reporting the superiority of the off-clamp technique in RAPN (9, 10, 12, 20), especially in patients who require preservation of renal function (e.g., solitary kidney or chronic kidney disease) (23). More well-designed, large-scale prospective randomized studies with long-term follow-up are needed to further compare the perioperative, oncological, and renal functional superiority in these two clamping approaches in RAPN.

## Conclusion

Pooled analyses demonstrated that off-clamp was an effective and safe technique with superiority in operating time, EBL, complications, positive surgical margins, and long-term preservation of renal function compared with the on-clamp approach in RAPN. Given the presence of heterogeneity and potential bias, urologists should select the clamp strategy based on their experience and patient-specific factors.

## Data Availability Statement

The raw data supporting the conclusions of this article will be made available by the authors, without undue reservation.

## Ethics Statement

Ethical review and approval were not required for the study on human participants in accordance with the local legislation and institutional requirements.

## Author Contributions

(I) Conception and design: YH and DC. (II) Administrative support: QD, LL, and QW. (III) Provision of study materials or patients: YH and DC. (IV) Collection and assembly of data: YH, DC, ZC, BC, JG, and JL. (V) Data analysis and interpretation: YH and DC. (VI) Manuscript writing: YH, DC, ZC, BC, JL, JG, QD, LL, and QW. All authors contributed to the article and approved the submitted version.

## Funding

This study was funded by the National Natural Science Foundation of China (Grant Number 82000721) and Programs from the Department of Science and Technology of Sichuan Province (Grant Number 2020YJ0054).

## Conflict of Interest

The authors declare that the research was conducted in the absence of any commercial or financial relationships that could be construed as a potential conflict of interest.

## Publisher’s Note

All claims expressed in this article are solely those of the authors and do not necessarily represent those of their affiliated organizations, or those of the publisher, the editors and the reviewers. Any product that may be evaluated in this article, or claim that may be made by its manufacturer, is not guaranteed or endorsed by the publisher.
