# Supplementary material for: Comparison of Perioperative, Renal Functional, and Oncological Outcomes Between Off-Clamp and On-Clamp Robot-Assisted Partial Nephrectomy for Renal Tumors: An Updated Evidence-Based Analysis
Source: Front Oncol. 2021 Sep 21;11:730662. doi: 10.3389/fonc.2021.730662 (PMC8490928; doi:10.3389/fonc.2021.730662)
Supplement: Supplementary file 1 [file DataSheet_1.zip › Supplementary Table 2.DOCX]

| Supplementary Table S2. Detailed search strategy in three databases. | |
| --- | --- |
| Database | Search strategy |
| Pubmed | (((((clamp) OR (clamping)) OR (off-clamp)) OR (on-clamp)) AND ((partial nephrectomy) OR (nephron sparing surgery))) AND ((((robot-assisted) OR (robotic-assisted)) OR (robot)) OR (robotic)) |
| Embase^*^ | 1 (robot-assisted or robotic-assisted or robot or robotic).af.  2 (partial nephrectomy or nephron sparing surgery).af.  3 (clamping or clamp or on-clamp or off-clamp).af.  4 1 and 2 and 3 |
| Web of Science | 1 robot-assisted (Topic) or robotic-assisted (Topic) or robot (Topic) or robotic (Topic)  2 clamp (Topic) or clamping (Topic) or off-clamp (Topic) or on-clamp (Topic)  3 clamp (Topic) or clamping (Topic) or off-clamp (Topic) or on-clamp (Topic)  4 ((#1) AND #2) AND #3 |
| ^*^ We retrieved articles from Embase via the Ovid (https://ovidsp.ovid.com/). | |
