# Supplementary material for: Comparison of Perioperative, Renal Functional, and Oncological Outcomes Between Off-Clamp and On-Clamp Robot-Assisted Partial Nephrectomy for Renal Tumors: An Updated Evidence-Based Analysis
Source: Front Oncol. 2021 Sep 21;11:730662. doi: 10.3389/fonc.2021.730662 (PMC8490928; doi:10.3389/fonc.2021.730662)
Supplement: Supplementary file 1 [file DataSheet_1.zip › Supplementary Table 3.DOCX]

| Supplementary Table S3. Quality evaluation of the eligible studies with Newcastle–Ottawa scale. | | | | | | | | | |
| --- | --- | --- | --- | --- | --- | --- | --- | --- | --- |
| Study | Selection | | | | Comparability | | Outcome | | |
|  | Representative-ness | Selection of  non-exposed | Ascertainment  of exposure | Outcome not present at start | Comparability on most important factors | Comparability on other risk factors | Assessment of outcome | Long enough follow-up (median≥1 year) | Adequacy  (completeness) of follow-up |
| White et al.([9](#_ENREF_9)) | * | * | * | * | * | - | * | * | * |
| Novak et al.([10](#_ENREF_10)) | * | * | * | * | - | - | * | - | * |
| Tanagho et al.([11](#_ENREF_11)) | * | * | * | * | * | - | * | - | * |
| Kaczmarek et al.([12](#_ENREF_12)) | * | * | * | * | * | - | * | * | * |
| Krane et al.([13](#_ENREF_13)) | * | * | * | * | - | - | * | - | * |
| Acar et al.([14](#_ENREF_14)) | * | * | * | * | * | - | * | * | * |
| Komninos et al.([15](#_ENREF_15)) | * | * | * | * | - | - | * | - | * |
| Ener et al.([16](#_ENREF_16)) | * | * | * | * | * | - | * | - | * |
| Peyronnet et al.([17](#_ENREF_17)) | * | * | * | * | * | - | * | * | * |
| Rosen et al.([18](#_ENREF_18)) | * | * | * | * | * | - | * | - | * |
| Anderson et al.([19](#_ENREF_19)) | * | * | * | * | * | - | * | - | * |
| Mari et al.([20](#_ENREF_20)) | * | * | * | * | * | - | * | * | * |
| Taweemonkongsap  et al.([21](#_ENREF_21)) | * | * | * | * | * | - | * | * | * |
| Anderson et al.([22](#_ENREF_22)) | * | * | * | * | * | - | * | - | * |
| Bertolo et al.([23](#_ENREF_23)) | * | * | * | * | * | - | * | - | * |
| Guo et al.([24](#_ENREF_24)) | * | * | * | * | * | - | * | * | * |
| Anceschi et al.([25](#_ENREF_25)) | - | - | * | * | * | - | * | * | * |
| Antonelli et al.([26](#_ENREF_26)) | * | * | * | * | - | - | * | - | * |
| Beksac et al.([27](#_ENREF_27)) | - | - | * | * | * | - | * | - | * |
| Antonelli et al.([28](#_ENREF_28)) | * | * | * | * | * | - | * | - | * |
| Mellouki et al.([29](#_ENREF_29)) | * | * | * | * | - | - | * | * | * |
| *indicates criterion met; - indicates significant of criterion not met. | | | | | | | | | |
